# Supplementary material for: Perineuronal Net Receptor PTPσ Regulates Retention of Memories
Source: Front Synaptic Neurosci. 2021 Jul 22;13:672475. doi: 10.3389/fnsyn.2021.672475 (PMC8339997; doi:10.3389/fnsyn.2021.672475)
Supplement: Supplementary file 1 [file Data_Sheet_1.PDF]

Supplementary figure 1. Immunohistochemical analysis of synapses in the brain of PTPσ$^{+/-}$ mice and their WT littermates.

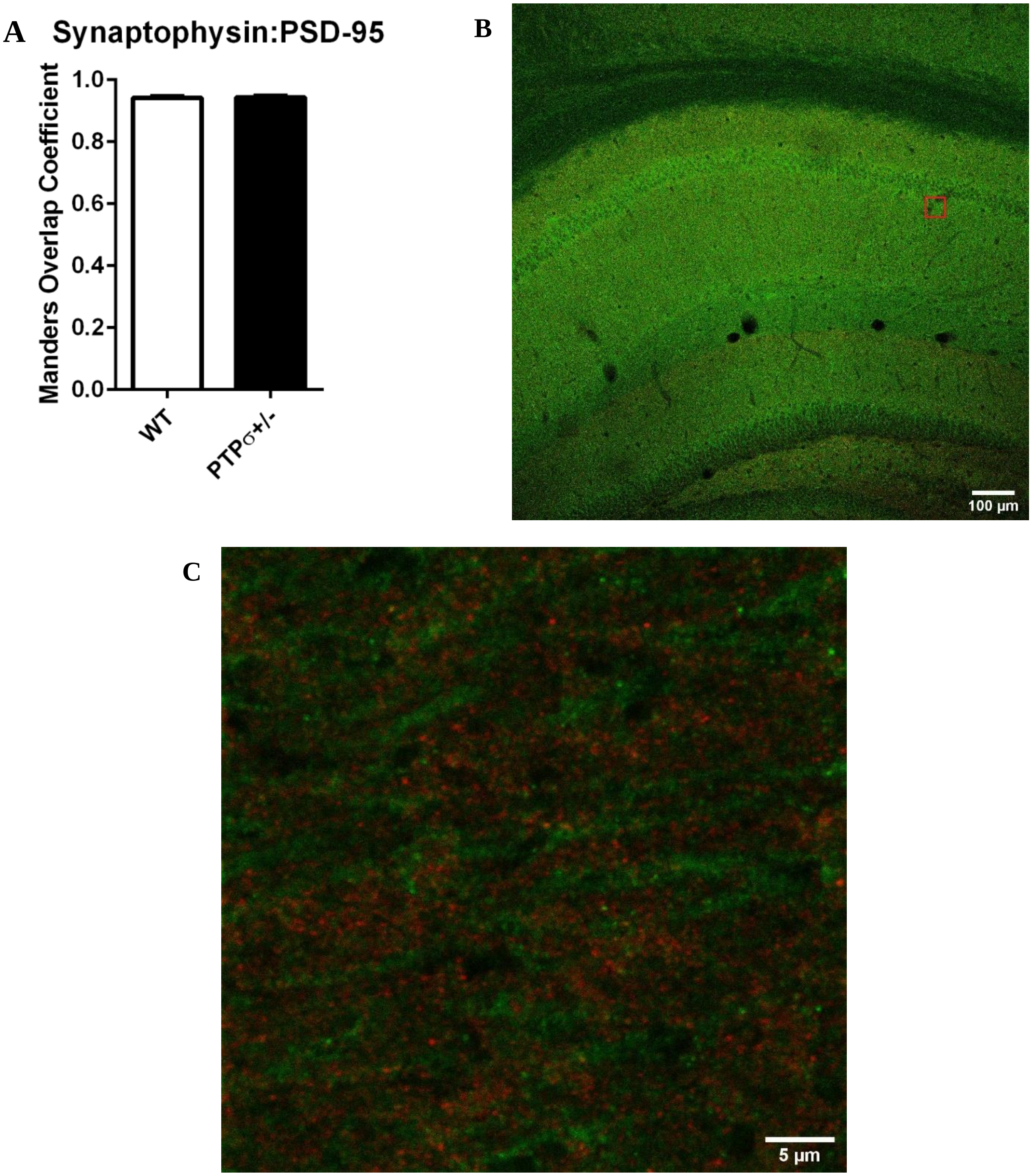

1 A. PTPσ$^{+/-}$ mice have similar number of excitatory synapses in the HPC as their WT littermates, as evaluated by colocalization analysis of Synaptophysin and PSD-95 expression using Mander's Overlap Coefficient. Analysis was done in the substratum radiatum of CA1 region where Schaffer collateral axons make synapses with CA1 neurons. Data were analyzed using t-test: $t(15) = 0.5888$, $p = 0.5648$ and presented as mean ± SD. Number of samples: WT: 10, PTPσ+/-: 7. **1 B.** Localization of the region where the imaging took place (red box). **1 C**. A representative image of Synaptophysin and PSD-95 staining. Red: synaptophysin, green: PSD-95.

Supplementary figure 2. Images of Western blot analysis of prefrontal cortex (PFC), hippocampus (HPC) and amygdala (AMG) brain samples from PTPσ$^{+/-}$ mice and their wild-type (WT) littermates. M – male, F – female samples.

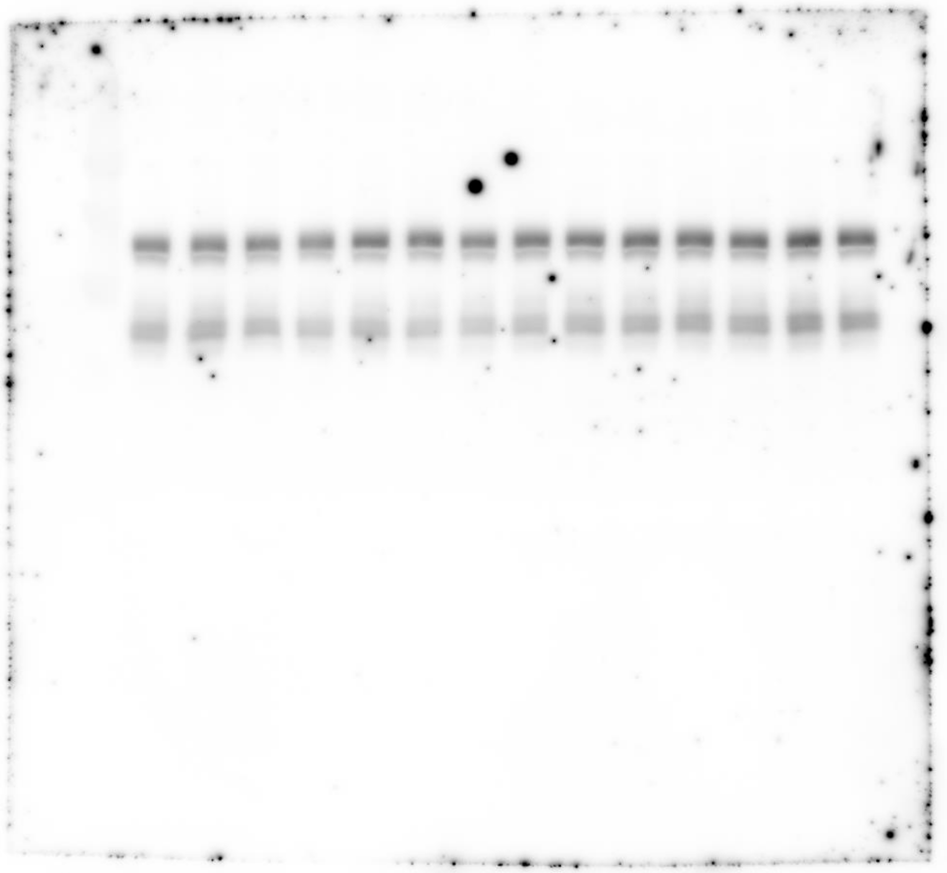

← 140 kDa

2 A. Total TRKB PFC. Samples from left to right:

M	F	M	M	M	F	M	M	M	F	M	F	F	M
PTPσ$^{+/-}$	WT	PTPσ$^{+/-}$	WT	PTPσ$^{+/-}$	PTPσ$^{+/-}$	WT	PTPσ$^{+/-}$	WT	PTPσ$^{+/-}$	PTPσ$^{+/-}$	WT	PTPσ$^{+/-}$	PTPσ$^{+/-}$

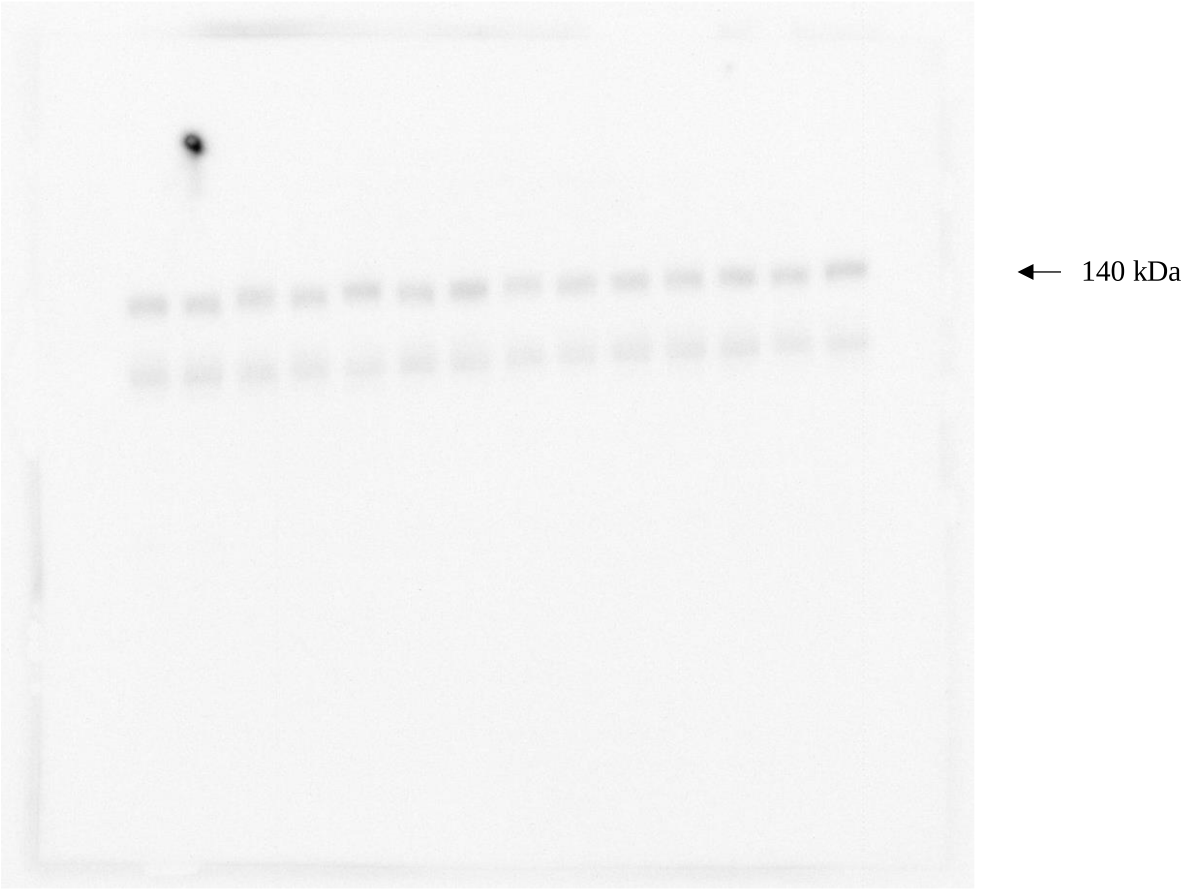

2 B. Total TRKB HPC. Samples from left to right:

M	F	M	M	M	F	M	M	M	F	M	F	F	M
PTPσ$^{+/-}$	WT	PTPσ$^{+/-}$	WT	PTPσ$^{+/-}$	PTPσ$^{+/-}$	WT	PTPσ$^{+/-}$	WT	PTPσ$^{+/-}$	PTPσ$^{+/-}$	WT	PTPσ$^{+/-}$	PTPσ$^{+/-}$

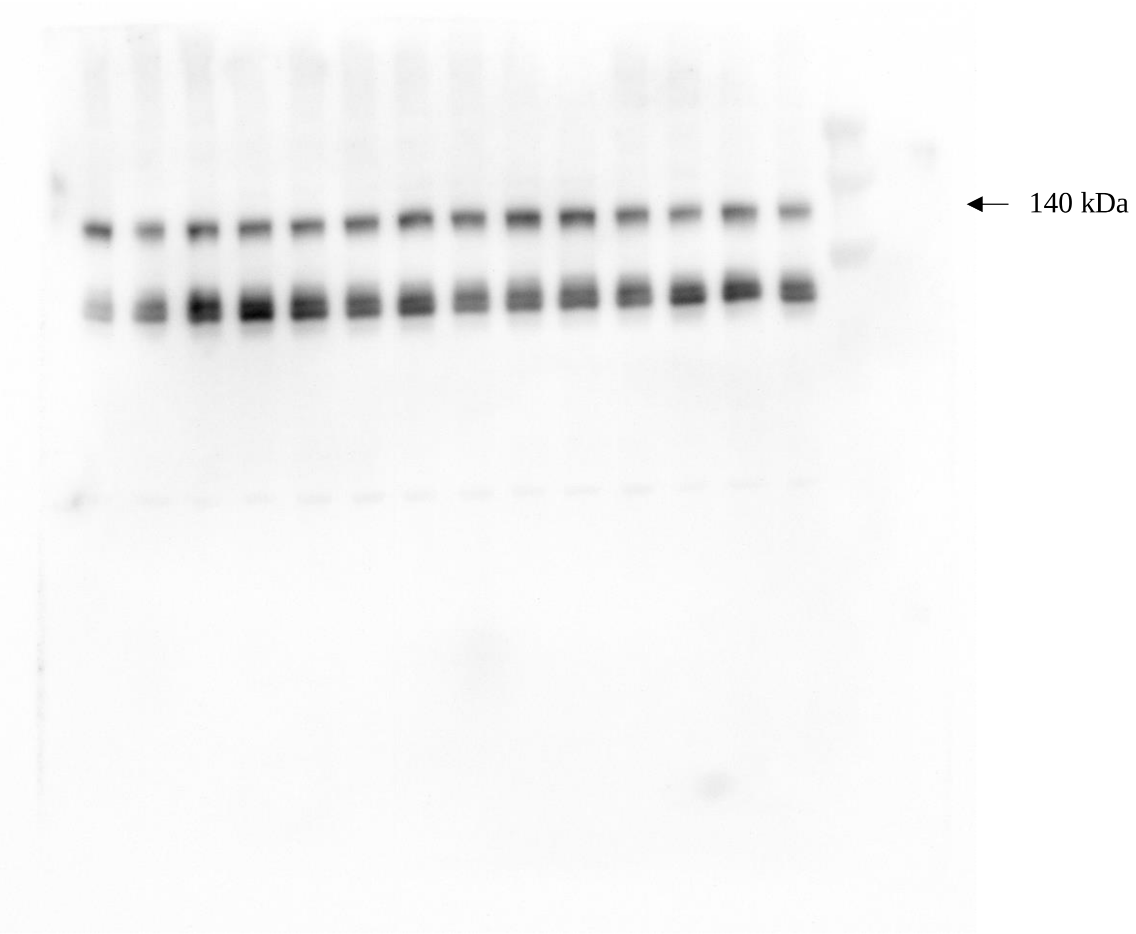

2 C. Total TRKB AMG. Samples from left to right:

F	F	M	M	M	F	M	M	M	F	M	F	F	M
PTPσ$^{+/-}$	WT	PTPσ$^{+/-}$	WT	PTPσ$^{+/-}$	PTPσ$^{+/-}$	WT	PTPσ$^{+/-}$	WT	PTPσ$^{+/-}$	PTPσ$^{+/-}$	WT	PTPσ$^{+/-}$	PTPσ$^{+/-}$

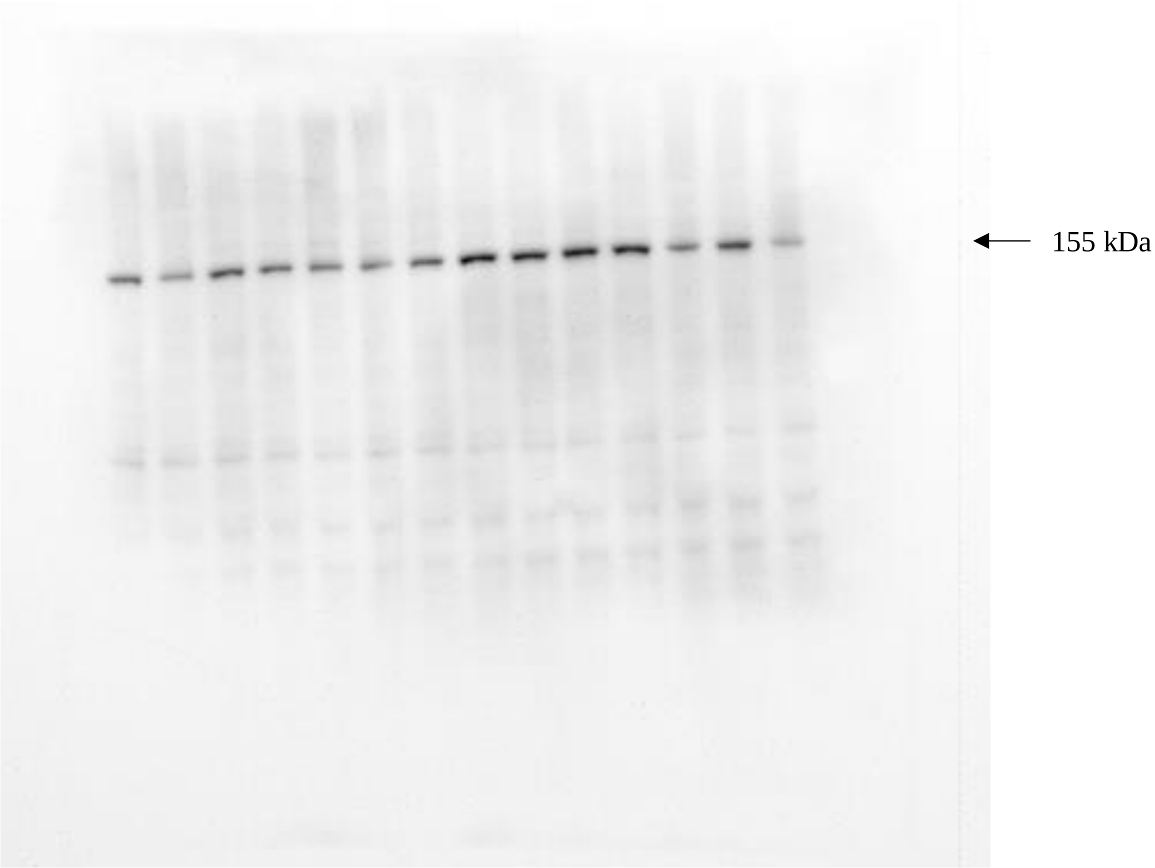

2 D. pPLCγ1 PFC. Samples from left to right:

M	F	M	M	M	F	M	M	M	F	M	F	F	M
PTPσ$^{+/-}$	WT	PTPσ$^{+/-}$	WT	PTPσ$^{+/-}$	PTPσ$^{+/-}$	WT	PTPσ$^{+/-}$	WT	PTPσ$^{+/-}$	PTPσ$^{+/-}$	WT	PTPσ$^{+/-}$	PTPσ$^{+/-}$

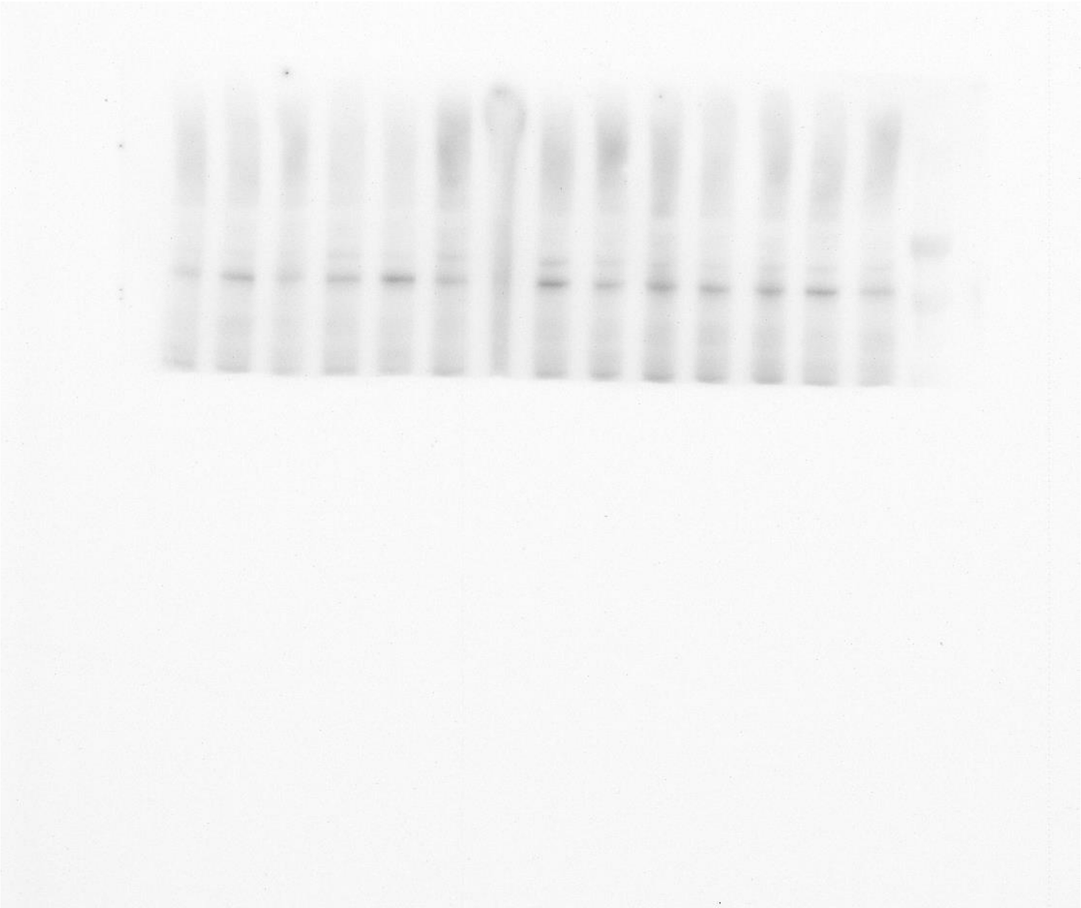

← 155 kDa

2 E. pPLCγ1 HPC. Samples from left to right:

M	F	M	M	M	F		M	M	F	M	F	F	M
$PTP\sigma^{+/-}$	WT	$PTP\sigma^{+/-}$	WT	$PTP\sigma^{+/-}$	$PTP\sigma^{+/-}$		$PTP\sigma^{+/-}$	WT	$PTP\sigma^{+/-}$	$PTP\sigma^{+/-}$	WT	$PTP\sigma^{+/-}$	$PTP\sigma^{+/-}$

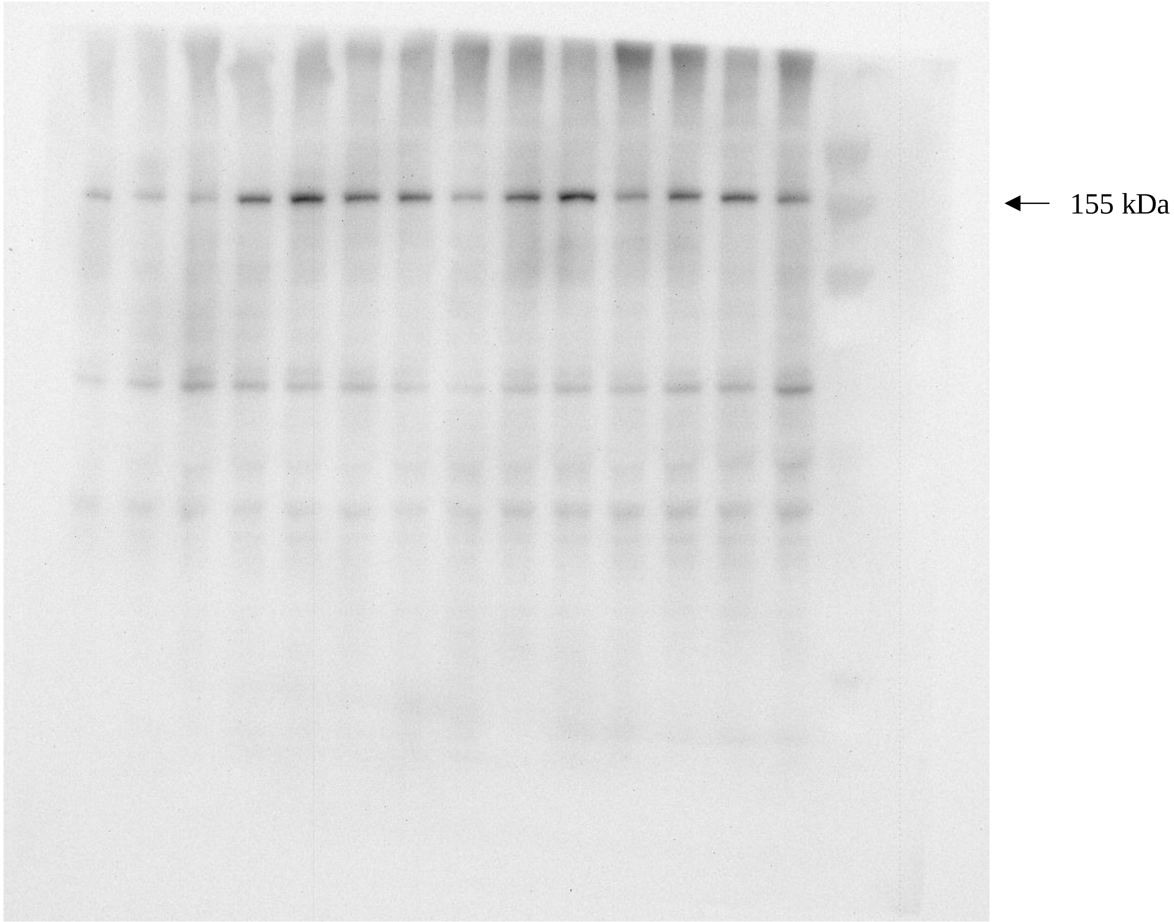

2 F. pPLCγ1 AMG. Samples from left to right:

F	F	M	M	M	F	M	M	M	F	M	F	F	M
PTPσ$^{+/-}$	WT	PTPσ$^{+/-}$	WT	PTPσ$^{+/-}$	PTPσ$^{+/-}$	WT	PTPσ$^{+/-}$	WT	PTPσ$^{+/-}$	PTPσ$^{+/-}$	WT	PTPσ$^{+/-}$	PTPσ$^{+/-}$

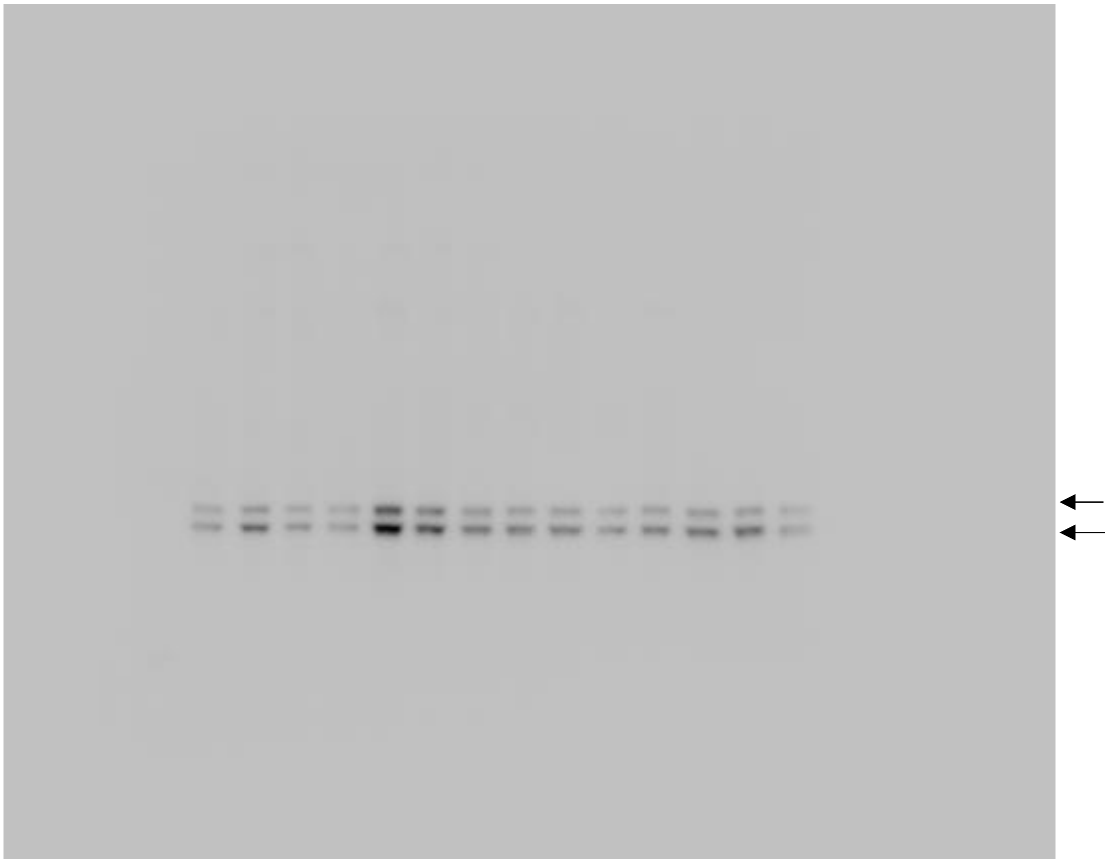

2 G. pErk PFC. Samples from left to right:

M	F	M	M	M	F	M	M	M	F	M	F	F	M
PTPσ$^{+/-}$	WT	PTPσ$^{+/-}$	WT	PTPσ$^{+/-}$	PTPσ$^{+/-}$	WT	PTPσ$^{+/-}$	WT	PTPσ$^{+/-}$	PTPσ$^{+/-}$	WT	PTPσ$^{+/-}$	PTPσ$^{+/-}$

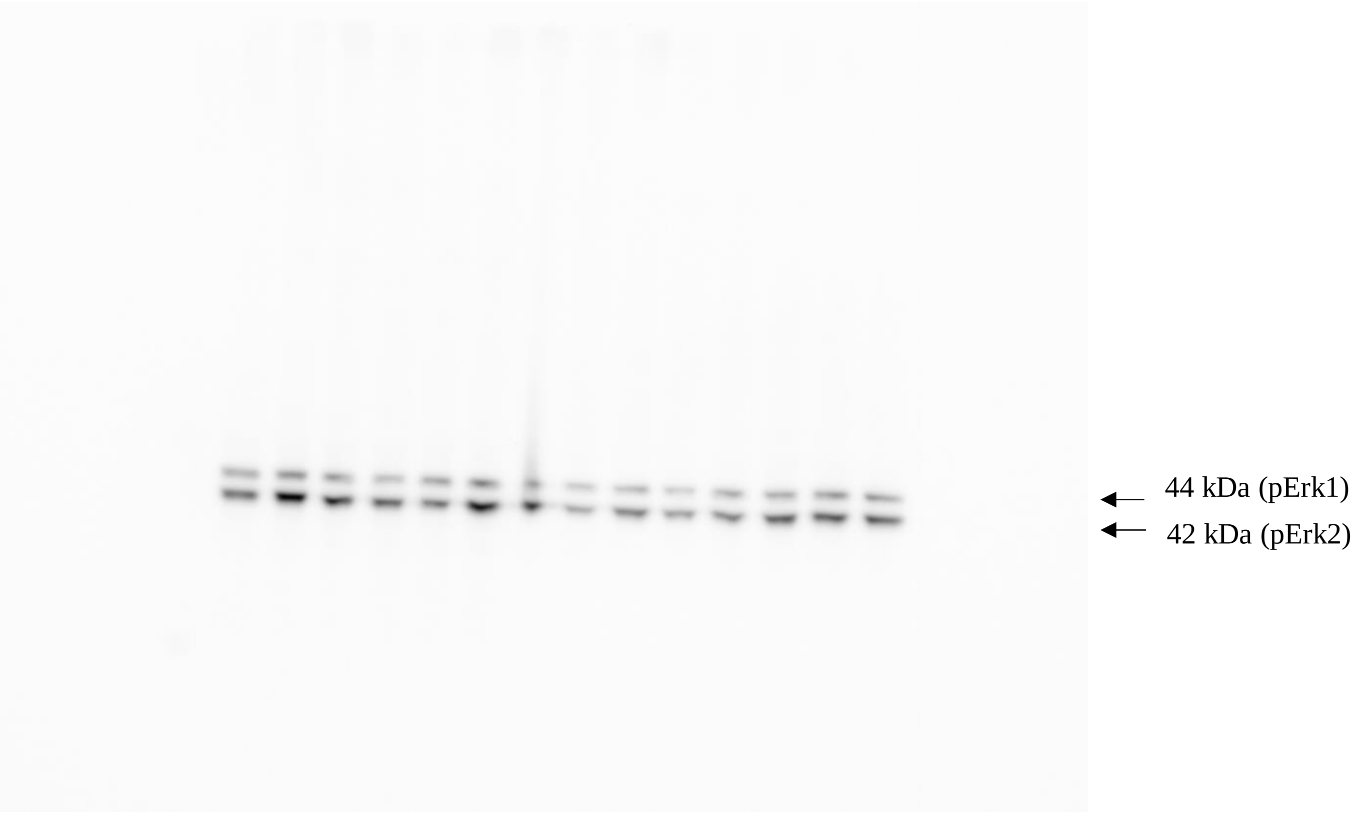

2 H. pErk HPC. Samples from left to right:

M	F	M	M	M	F	M	M	M	F	M	F	F	M
PTPσ$^{+/-}$	WT	PTPσ$^{+/-}$	WT	PTPσ$^{+/-}$	PTPσ$^{+/-}$	WT	PTPσ$^{+/-}$	WT	PTPσ$^{+/-}$	PTPσ$^{+/-}$	WT	PTPσ$^{+/-}$	PTPσ$^{+/-}$

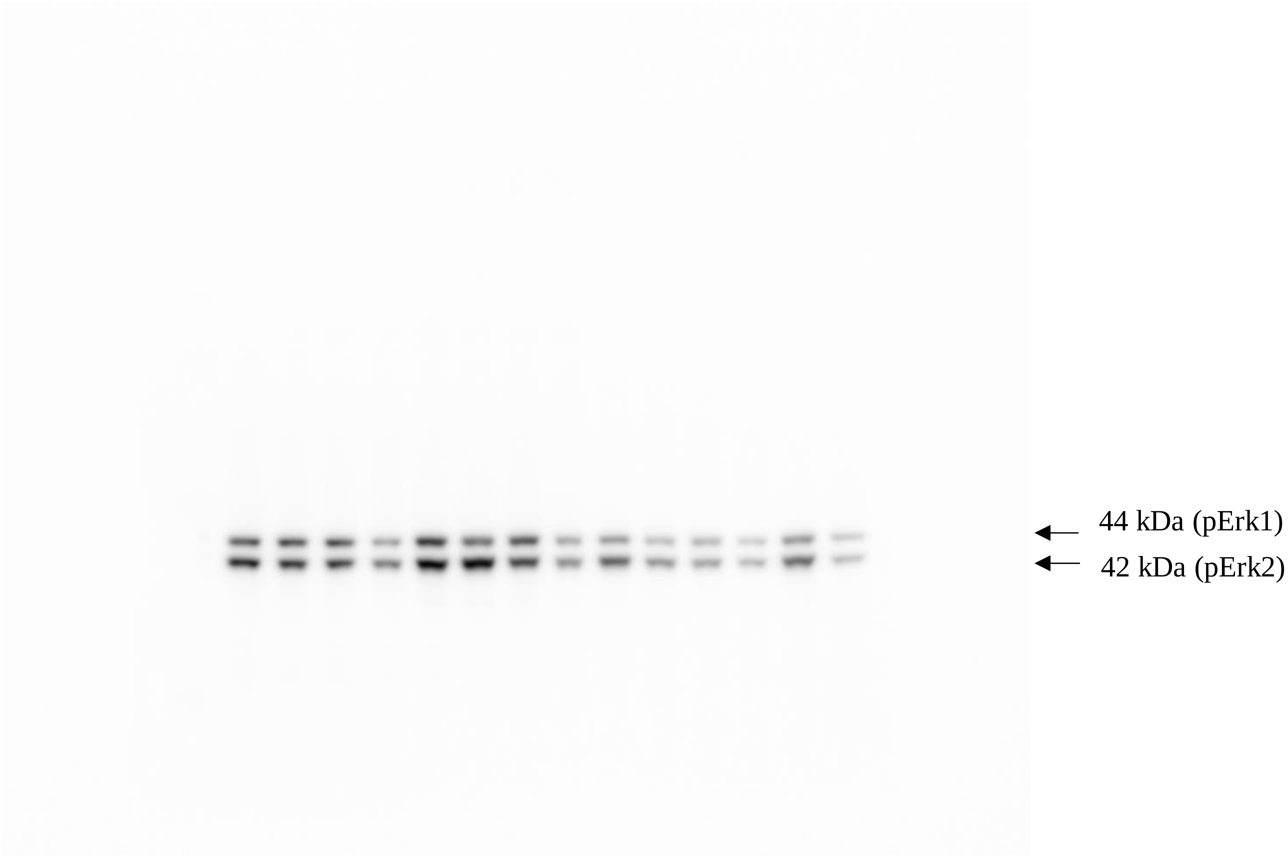

2 I. pErk AMG. Samples from left to right:

F	F	M	M	M	F	M	M	M	F	M	F	F	M
PTPσ$^{+/-}$	WT	PTPσ$^{+/-}$	WT	PTPσ$^{+/-}$	PTPσ$^{+/-}$	WT	PTPσ$^{+/-}$	WT	PTPσ$^{+/-}$	PTPσ$^{+/-}$	WT	PTPσ$^{+/-}$	PTPσ$^{+/-}$

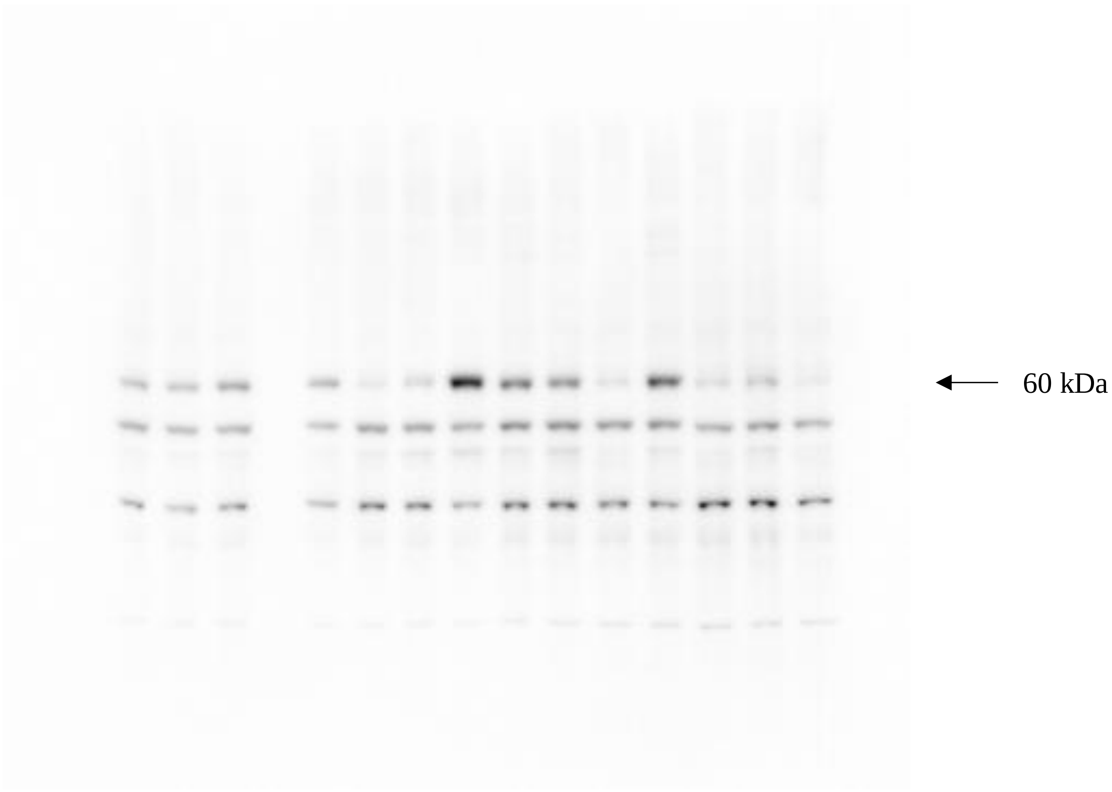

2 J. pAkt PFC. Samples from left to right:

M	F	M	M	F	M	M	M	M	F	M	F	F	M
PTPσ$^{+/-}$	WT	PTPσ$^{+/-}$	WT	PTPσ$^{+/-}$	PTPσ$^{+/-}$	WT	PTPσ$^{+/-}$	WT	PTPσ$^{+/-}$	PTPσ$^{+/-}$	WT	PTPσ$^{+/-}$	PTPσ$^{+/-}$

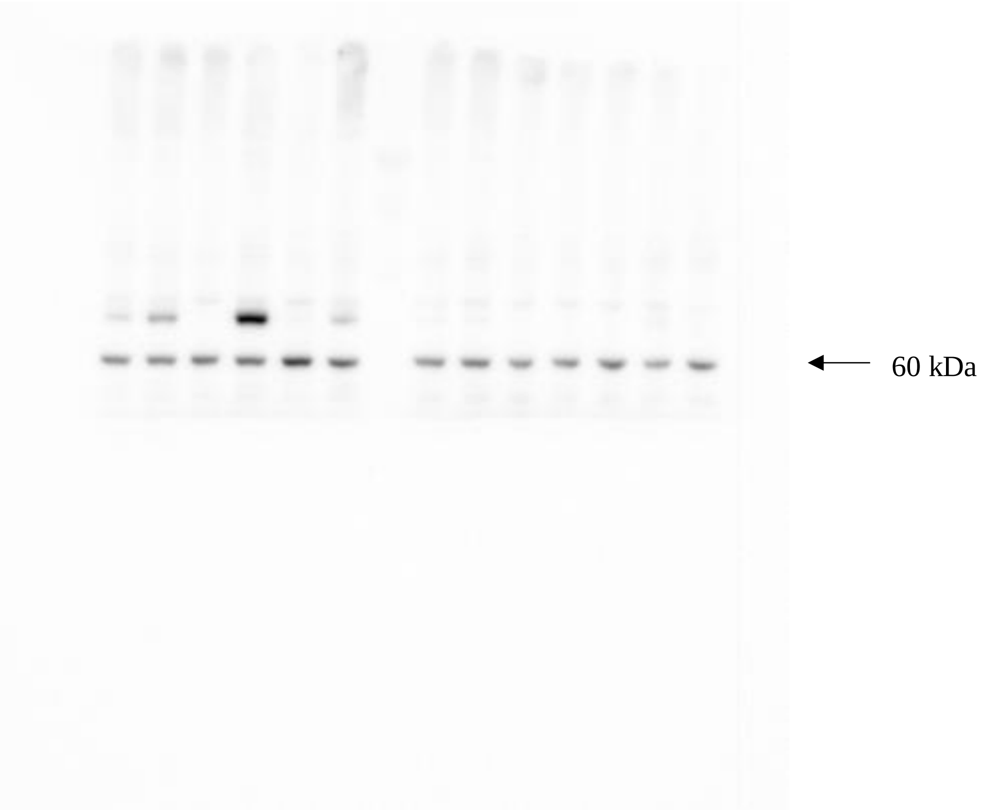

2 K. pAkt HPC. Lower part of the membrane was covered during imaging to avoid strong signal from non-specific binding interfering with the quality of the specific signal. Samples from left to right:

M	F	M	M	M	F	M	M	F	M	F	F	M
PTPσ$^{+/-}$	WT	PTPσ$^{+/-}$	WT	PTPσ$^{+/-}$	PTPσ$^{+/-}$	PTPσ$^{+/-}$	WT	PTPσ$^{+/-}$	PTPσ$^{+/-}$	WT	PTPσ$^{+/-}$	PTPσ$^{+/-}$

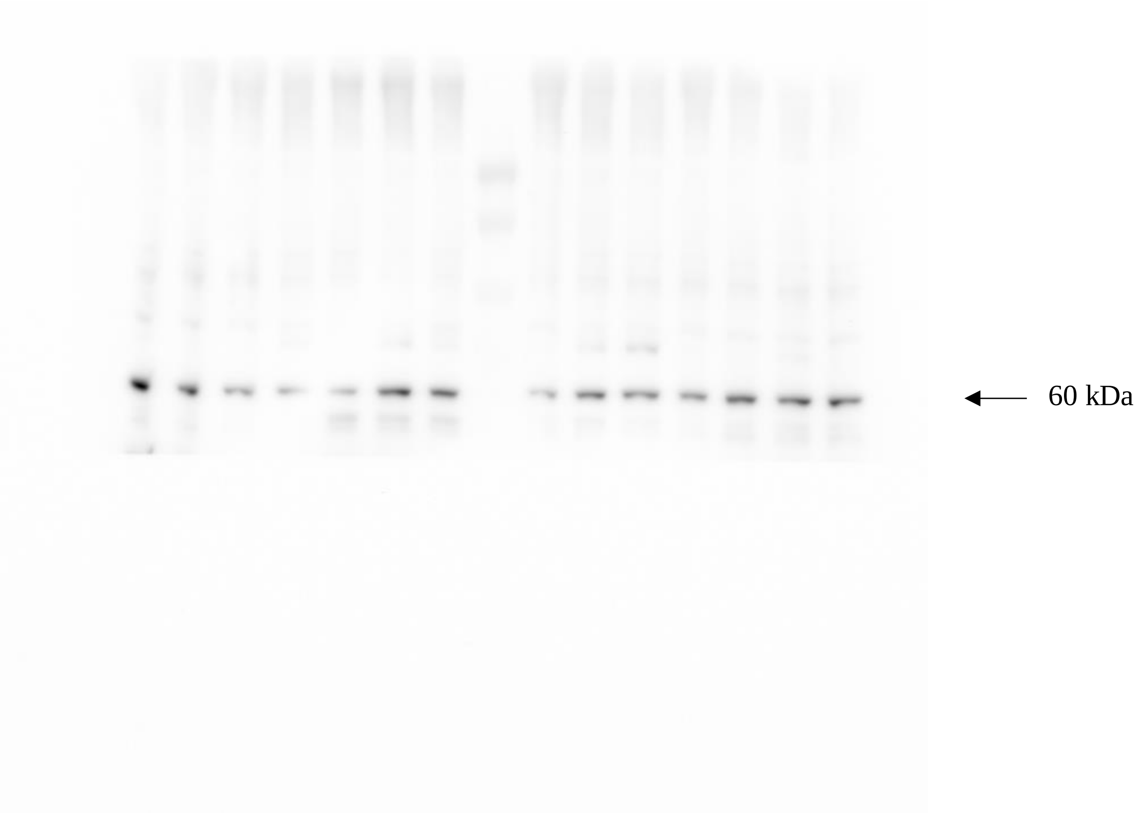

2 L. pAkt AMG. Lower part of the membrane was covered during imaging to avoid strong signal from non-specific binding interfering with the quality of the specific signal. Samples from left to right:

F	F	M	M	M	F	M	M	M	F	M	F	F	M
PTPσ$^{+/-}$	WT	PTPσ$^{+/-}$	WT	PTPσ$^{+/-}$	PTPσ$^{+/-}$	WT	PTPσ$^{+/-}$	WT	PTPσ$^{+/-}$	PTPσ$^{+/-}$	WT	PTPσ$^{+/-}$	PTPσ$^{+/-}$

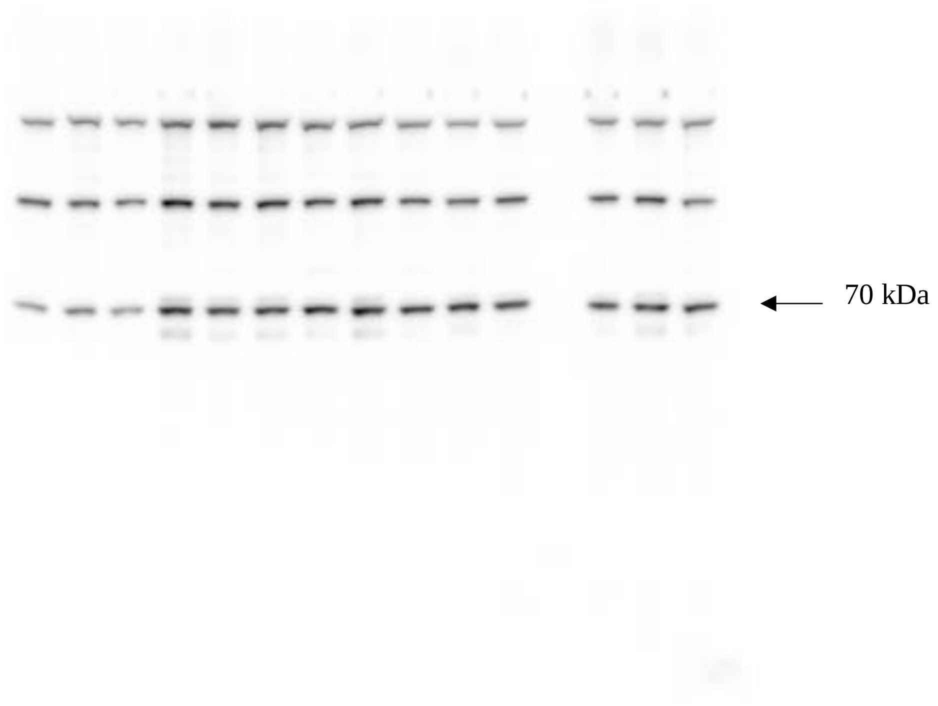

2 M. p-p70S6K PFC. Samples from left to right:

F	M	M	M	F	M	M	M	F	M	F	F	M	M
WT	PTPσ$^{+/-}$	WT	PTPσ$^{+/-}$	PTPσ$^{+/-}$	WT	PTPσ$^{+/-}$	WT	PTPσ$^{+/-}$	PTPσ$^{+/-}$	WT	PTPσ$^{+/-}$	PTPσ$^{+/-}$	PTPσ$^{+/-}$

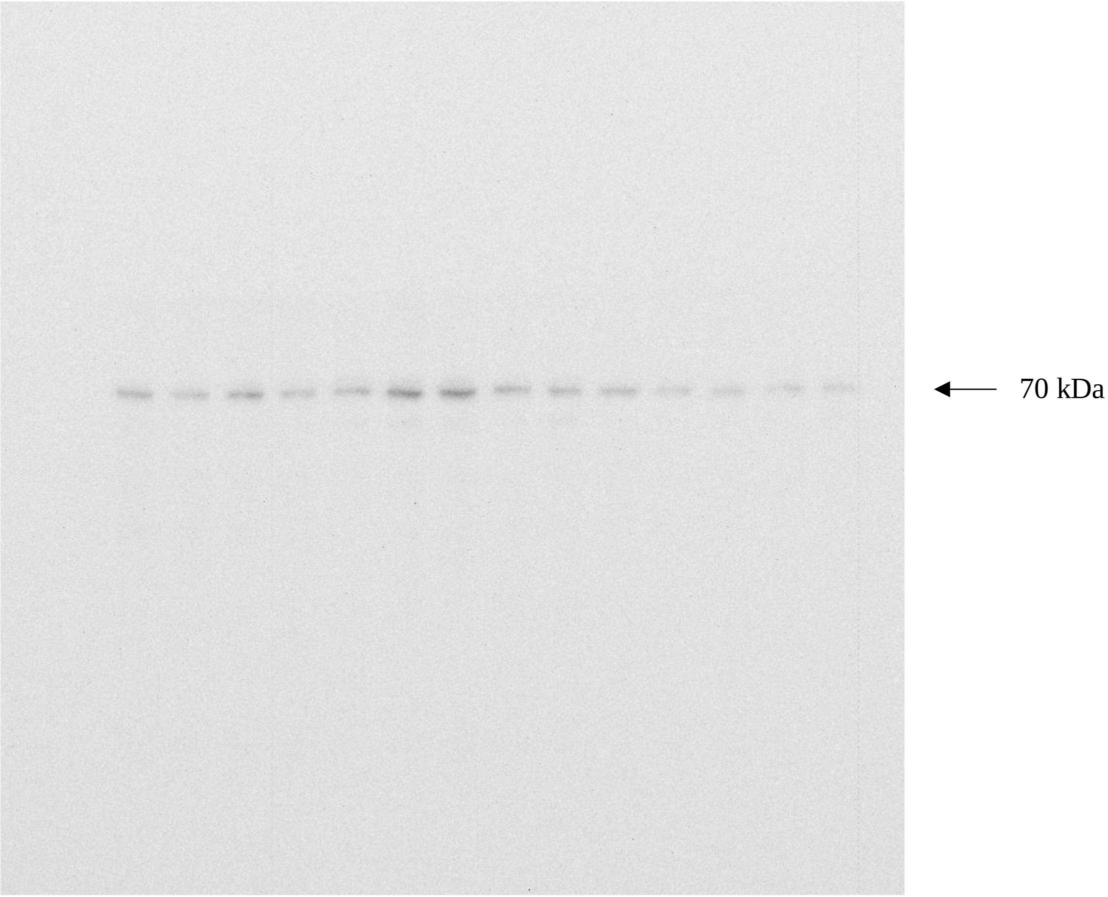

2 N. p-p70S6K HPC. Upper part of the membrane was covered during imaging to avoid strong signal from non-specific binding interfering with the quality of the specific signal. Samples from left to right:

F	F	M	M	M	F	M	M	F	M	F	F	M
$PTP\sigma^{+/-}$	WT	$PTP\sigma^{+/-}$	WT	$PTP\sigma^{+/-}$	$PTP\sigma^{+/-}$	$PTP\sigma^{+/-}$	WT	$PTP\sigma^{+/-}$	$PTP\sigma^{+/-}$	WT	$PTP\sigma^{+/-}$	$PTP\sigma^{+/-}$

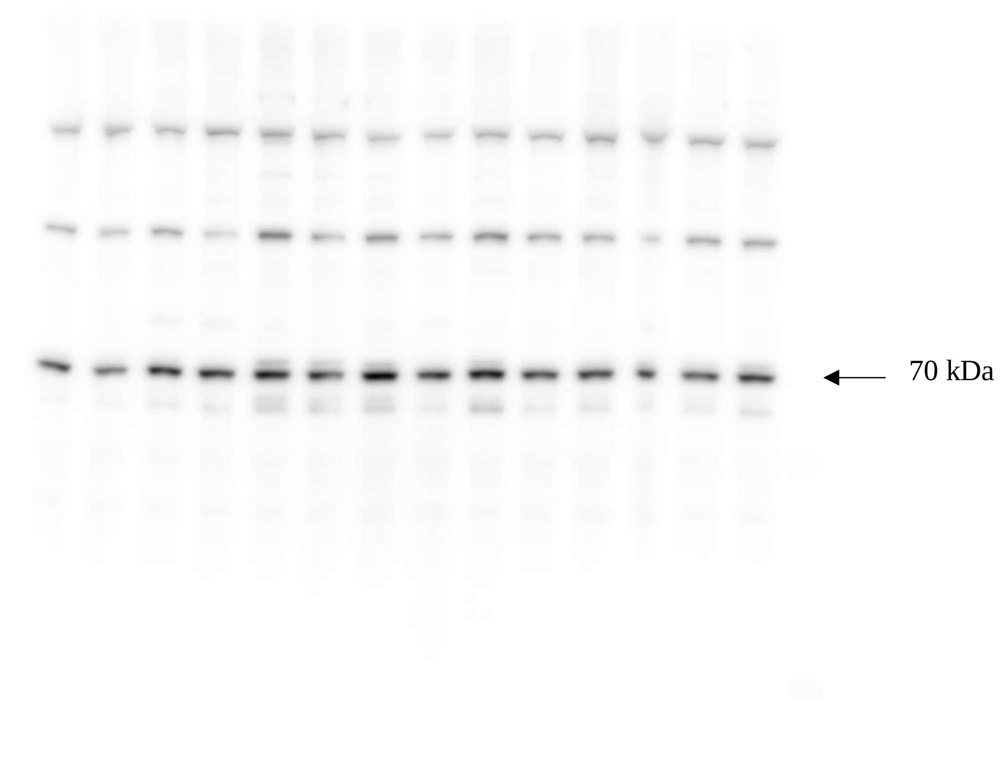

2 O. p-p70S6K AMG. Samples from left to right:

F	F	M	M	M	F	M	M	M	F	M	F	F	M
PTPσ$^{+/-}$	WT	PTPσ$^{+/-}$	WT	PTPσ$^{+/-}$	PTPσ$^{+/-}$	WT	PTPσ$^{+/-}$	WT	PTPσ$^{+/-}$	PTPσ$^{+/-}$	WT	PTPσ$^{+/-}$	PTPσ$^{+/-}$

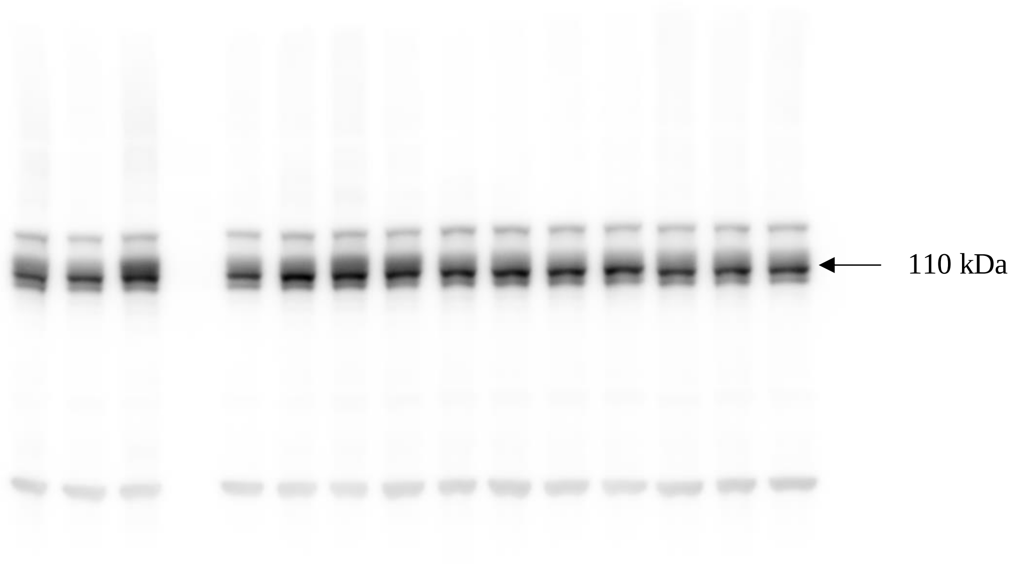

2 P. PSD-93 PFC. Samples from left to right:

M	F	M	M	F	M	M	M	M	F	M	F	F	M
PTPσ$^{+/-}$	WT	PTPσ$^{+/-}$	WT	PTPσ$^{+/-}$	PTPσ$^{+/-}$	WT	PTPσ$^{+/-}$	WT	PTPσ$^{+/-}$	PTPσ$^{+/-}$	WT	PTPσ$^{+/-}$	PTPσ$^{+/-}$

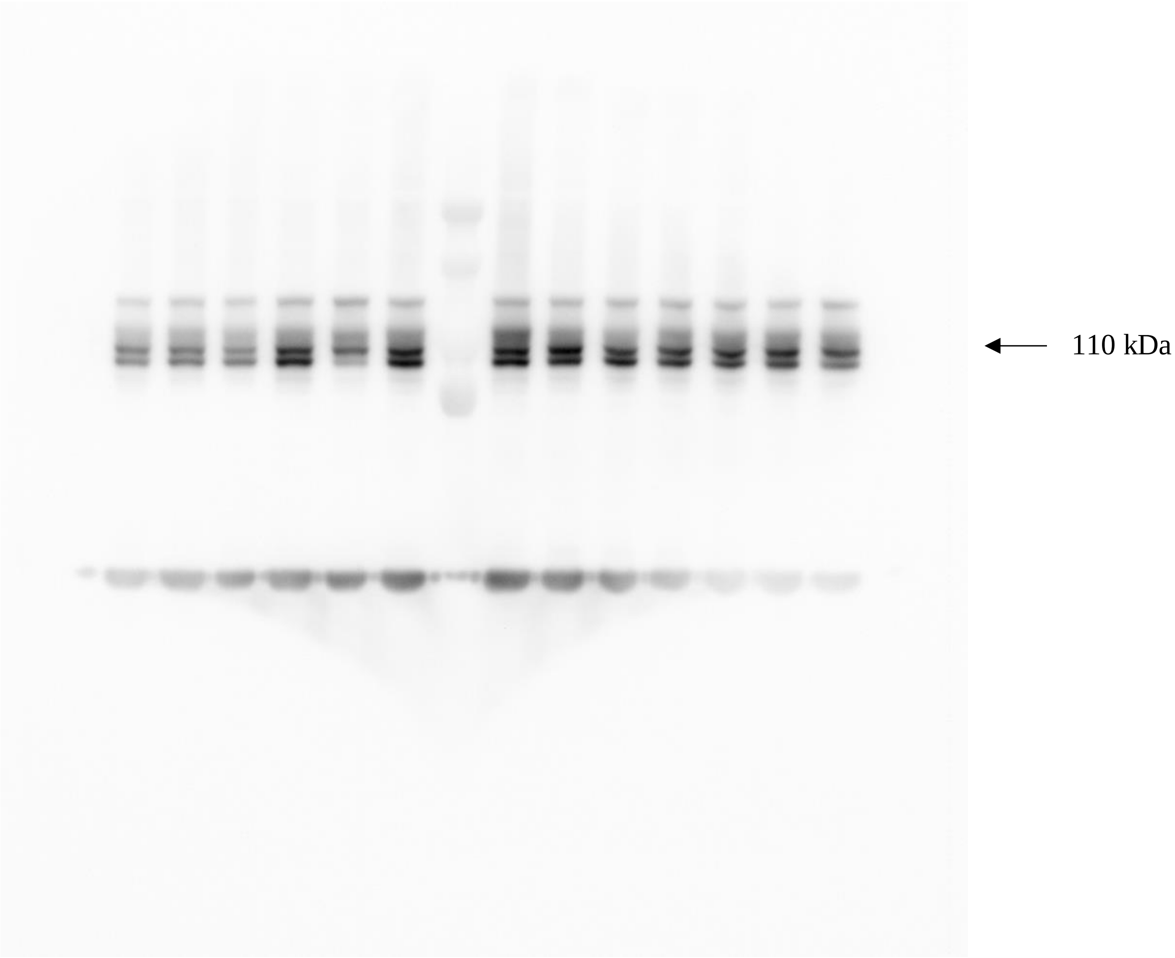

2 Q. PSD-93 HPC. Samples from left to right:

M	F	M	M	M	F	M	M	F	M	F	F	M
PTPσ$^{+/-}$	WT	PTPσ$^{+/-}$	WT	PTPσ$^{+/-}$	PTPσ$^{+/-}$	PTPσ$^{+/-}$	WT	PTPσ$^{+/-}$	PTPσ$^{+/-}$	WT	PTPσ$^{+/-}$	PTPσ$^{+/-}$

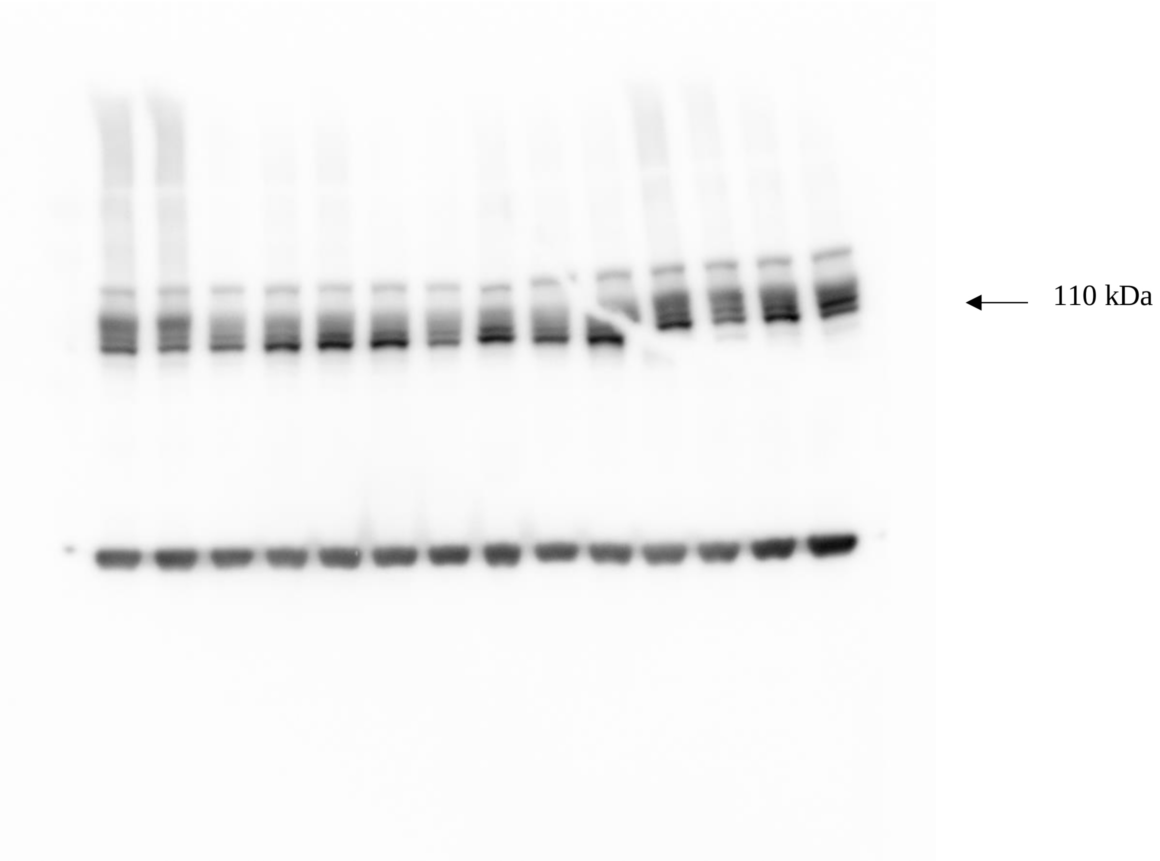

2 R. PSD-93 AMG. Samples from left to right:

F	F	M	M	M	F	M	M	M	F	M	F	F	M
PTPσ$^{+/-}$	WT	PTPσ$^{+/-}$	WT	PTPσ$^{+/-}$	PTPσ$^{+/-}$	WT	PTPσ$^{+/-}$	WT	PTPσ$^{+/-}$	PTPσ$^{+/-}$	WT	PTPσ$^{+/-}$	PTPσ$^{+/-}$

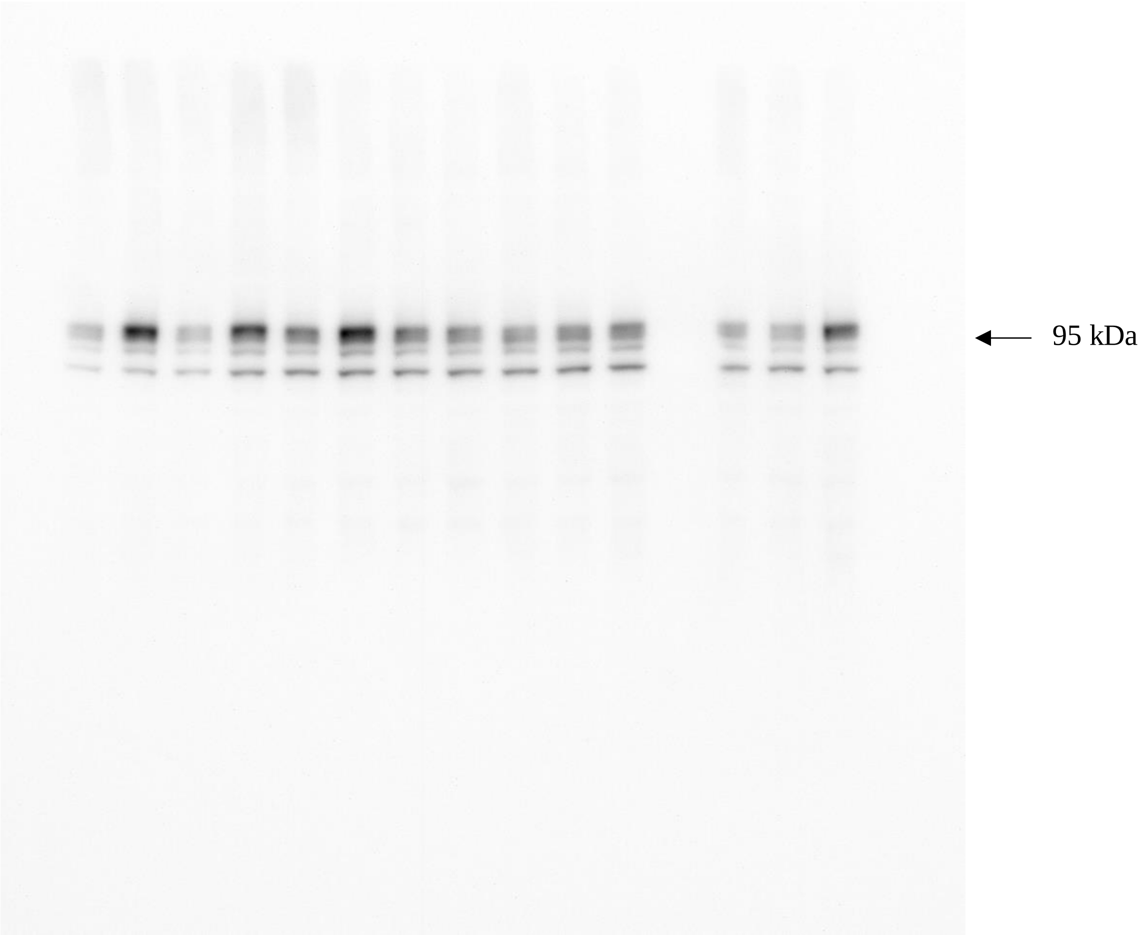

2 S. PSD-95 PFC. Samples from left to right:

M	F	M	M	F	M	M	M	M	F	M	F	F	M
$PTP\sigma^{+/-}$	WT	$PTP\sigma^{+/-}$	WT	$PTP\sigma^{+/-}$	$PTP\sigma^{+/-}$	WT	$PTP\sigma^{+/-}$	WT	$PTP\sigma^{+/-}$	$PTP\sigma^{+/-}$	WT	$PTP\sigma^{+/-}$	$PTP\sigma^{+/-}$

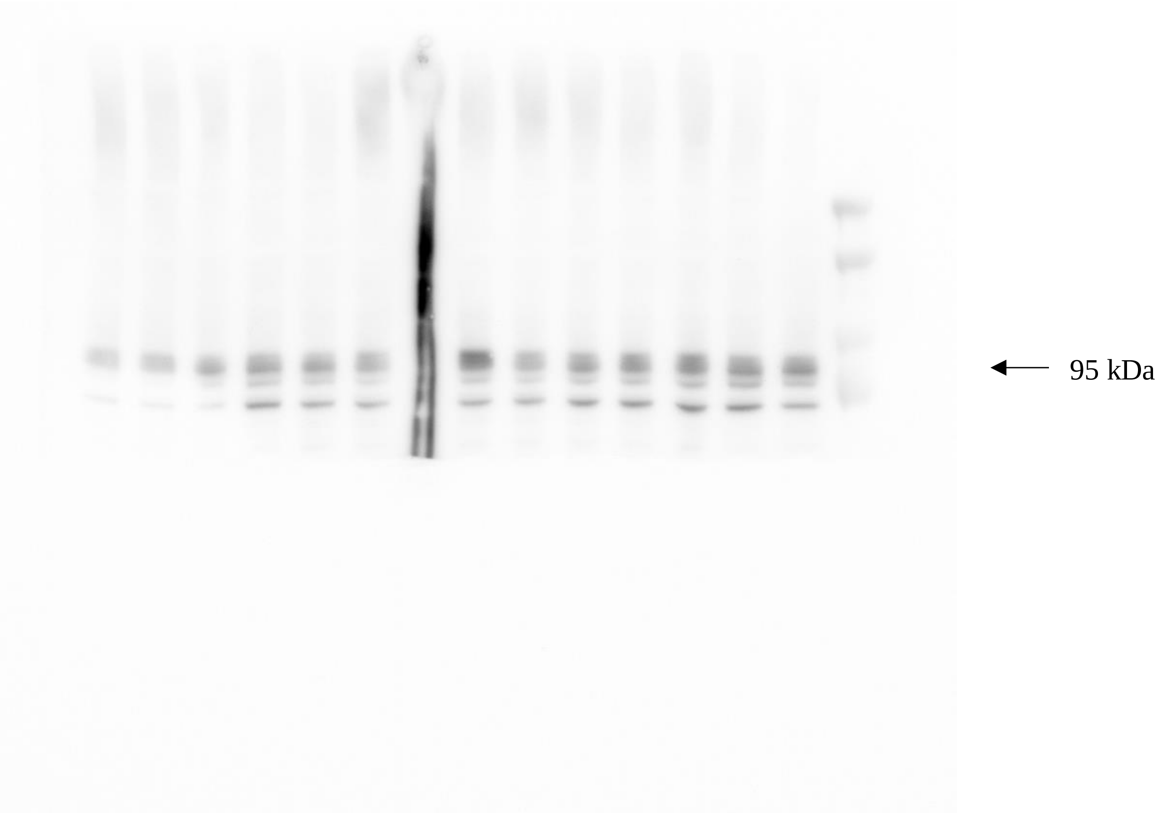

2 T. PSD-95 HPC. Samples from left to right:

M	F	M	M	M	F		M	M	F	M	F	F	M
PTPσ$^{+/-}$	WT	PTPσ$^{+/-}$	WT	PTPσ$^{+/-}$	PTPσ$^{+/-}$		PTPσ$^{+/-}$	WT	PTPσ$^{+/-}$	PTPσ$^{+/-}$	WT	PTPσ$^{+/-}$	PTPσ$^{+/-}$

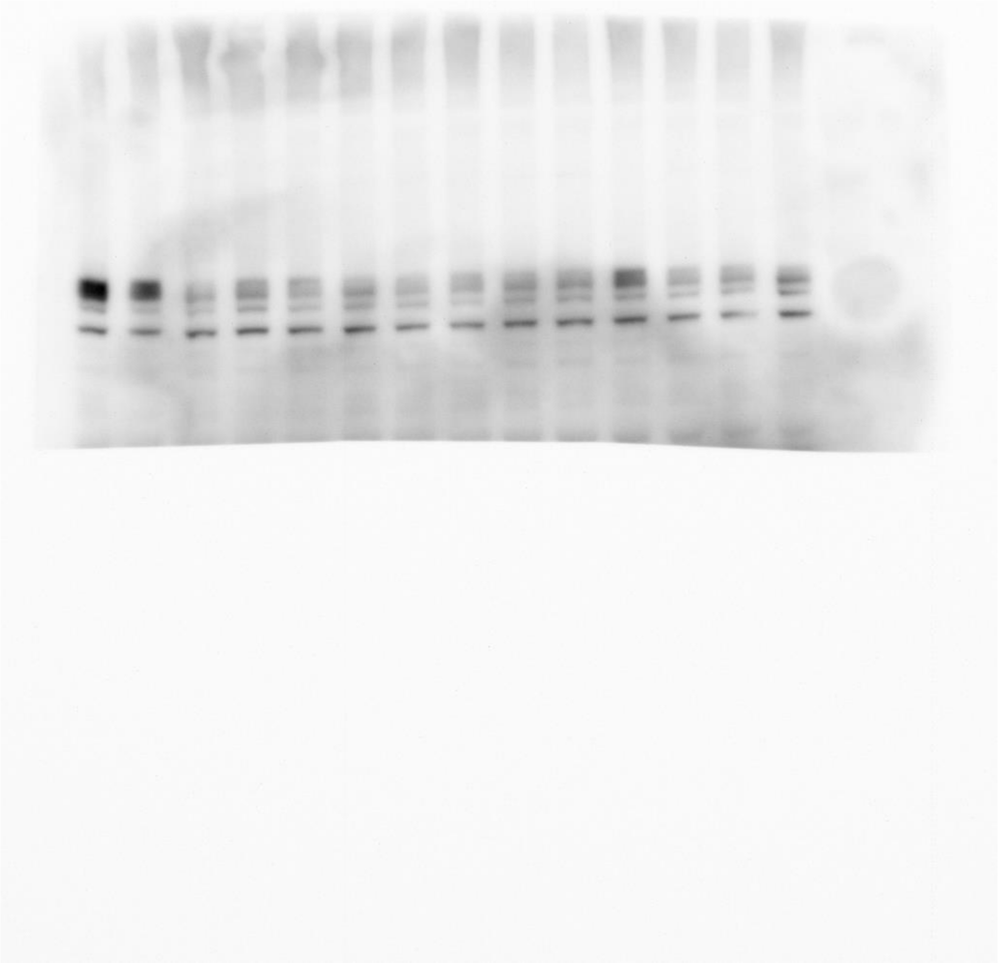

95 kDa

2 U. PSD-95 AMG. Samples from left to right:

F	F	M	M	M	F	M	M	M	F	M	F	F	M
PTPσ$^{+/-}$	WT	PTPσ$^{+/-}$	WT	PTPσ$^{+/-}$	PTPσ$^{+/-}$	WT	PTPσ$^{+/-}$	WT	PTPσ$^{+/-}$	PTPσ$^{+/-}$	WT	PTPσ$^{+/-}$	PTPσ$^{+/-}$
